# Retinol-Containing Graft Copolymers for Delivery of Skin-Curing Agents

**DOI:** 10.3390/pharmaceutics11080378

**Published:** 2019-08-02

**Authors:** Justyna Odrobińska, Katarzyna Niesyto, Karol Erfurt, Agnieszka Siewniak, Anna Mielańczyk, Dorota Neugebauer

**Affiliations:** 1 Department of Physical Chemistry and Technology of Polymers, Faculty of Chemistry, Silesian University of Technology, 44-100 Gliwice, Poland; 2 Department of Chemical Organic Technology and Petrochemistry, Faculty of Chemistry, Silesian University of Technology, 44-100 Gliwice, Poland

**Keywords:** retinol, “click” chemistry, alkyne–azide reaction, ATRP, graft copolymers, amphiphilic copolymers, micellar carriers

## Abstract

The new polymeric systems for delivery in cosmetology applications were prepared using self-assembling amphiphilic graft copolymers. The synthesis based on “click” chemistry reaction included grafting of azide-functionalized polyethylene glycol (PEG-N_3_) onto multifunctional polymethacrylates containing alkyne units. The latter ones were obtained via atom transfer radical polymerization (ATRP) of alkyne-functionalized monomers, e.g., ester of hexynoic acid and 2-hydroxyethyl methacrylate (AlHEMA) with methyl methacrylate (MMA), using bromoester-modified retinol (RETBr) as the initiator. Varying the content of alkyne moieties adjusted by initial monomer ratios of AlHEMA/MMA was advantageous for the achievement of a well-defined grafting degree. The designed amphiphilic graft copolymers P((HEMA-*graft*-PEG)-*co*-MMA), showing tendency to micellization in aqueous solution at room temperature, were encapsulated with arbutin (ARB) or vitamin C (VitC) with high efficiencies (>50%). In vitro experiments carried out in the phosphate-buffered saline solution (PBS) at pH 7.4 indicated the maximum release of ARB after at least 20 min and VitC within 10 min. The fast release of the selected antioxidants and skin-lightening agents by these micellar systems is satisfactory for applications in cosmetology, where they can be used as the components of masks, creams, and wraps.

## 1. Introduction

Innovative drug delivery systems (DDS) with polymeric carriers are designed to prolong and improve the action of biologically active substances, including pharmaceuticals, in the body, providing controlled and targeted therapies [1,2,3,4]. These polymers should be non-toxic, non-immunogenic, biocompatible with optional biodegradability, and chemically inert [5]. The well-fitted structures of well-defined polymers are synthesized to achieve the desired drug loading and release with efficient concentration at a proper time.

Biopolymers, such as chitosan [6,7], hyaluronic acid [8,9], collagen [10], or dextran [11], with the ability to aggregate, are often used as carriers in cosmetology. Among the synthesized amphiphilic polymers, the most common are those based on 2-hydroxyethyl methacrylate (HEMA) [12], *N*-isopropylacrylamide [13], 2-(diethylamino)ethyl methacrylate [14], methacrylic acid [15,16,17], and polyethylene glycol (PEG) [14,18,19,20]. These are mostly block copolymers [21], including star [22] and graft [23,24,25] topologies, obtained by the controlled polymerization methods for DDS applications. Specific graft copolymers [26] were achieved by combination of the backbone with side chains, which can be introduced by grafting from [27,28] and grafting through [29,30], or via a combination of both to attain block side chains [15], heterografted structures [31], or brush–block–brush [20].

The excellent biocompatibility, hydrophilicity, good blood compatibility, high water content, and permeability of the HEMA-based polymers [32] resulted in them finding numerous applications as biomaterials [33], for example, hydrogels [34], for manufacturing contact lenses with drug delivery [35], or artificial implants [36]. The presence of the hydroxyl group in HEMA is advantageous for modification using pre- or post-polymerization reactions. Pre-polymerization modification is usually performed to convert a hydroxyl group into another with particular properties, e.g., a bromoester group initiating an atom transfer radical polymerization (ATRP) reaction [37] or a trimethylsilyl protecting group [31,38]. Another opportunity is the preparation of azide-functionalized monomers [39] or amine-functionalized monomers via an alkyne–azide “click” chemistry reaction with the formation of a triazole ring [40]. However, the “click” approach is commonly applied after the polymerization, when the polymer is modified with “clickable” groups (post-polymerization modification). The introduction of alkyne groups into the polymer was helpful in further functionalization with specific groups, e.g., pentafluorobenzyl [41], or oligomers, e.g., polyhedral oligomeric silsesquioxanes [42], or to attach polymer chains, e.g., PEG-N_3_ (grafting onto) [43].

In our previous work, HEMA-based polymers were successfully synthesized with the use of bromoester-functionalized retinol as a novel ATRP bioinitiator [44]. Retinol (vitamin A) is a well-known factor which stimulates collagen and glycosaminoglycan synthesis, supporting the reduction of wrinkles, acne, and hyperpigmentation. The aim of the current work was to prepare the alkyne-functionalized HEMA-based polymers for the “click” chemistry reaction. The alkyne group was introduced into the HEMA monomer (AlHEMA) via the esterification of the hydroxyl group with hexynoic acid (pre-polymerization modification), whereas the retinol-initiated alkyne-functionalized polymethacrylates resulting from ATRP were modified by grafting onto via an alkyne–azide “click” reaction to obtain the amphiphilic copolymers with PEG side chains. The used strategy is different from that reported in the literature because it provided polymers with adjustable amounts of alkyne groups (by proper ratios of AlHEMA to comonomer) as the guaranteed “click” sites. The grafted copolymers with self-assembling abilities were also examined for the encapsulation of active substances for skin treatment, such as arbutin or vitamin C, to show delivery activities of these potential systems for cosmetology.

## 2. Experimental

### 2.1. Materials

Methyl methacrylate (MMA, 99%, Alfa Aesar, Warsaw, Poland), 2-hydroxyethyl methacrylate (HEMA, 97%, Aldrich, Poznań, Poland), and anisole (99%, Alfa Aesar) were dried over molecular sieves and stored in a freezer under nitrogen. Copper (I) bromide (CuBr, 98%, Fluka, Steinheim, Germany) was purified by stirring in glacial acetic acid, followed by filtration and washing with ethanol and diethyl ether. After that, the solids were dried under vacuum. Additionally, 4,4-dinonyl-2,2-dipyridyl (dNdpy, 97%, Aldrich), *N,N,N′,N″,N″*-pentamethyldiethylenetriamine (PMDTA, 98%, Aldrich), triethylamine (TEA, 99%, Aldrich), pyridine (99%, Aldrich), 2-bromoisobutyryl bromide (BriBuBr, 98%, Aldrich), ethyl α-bromoisobutyrate (EiB-Br, Aldrich, 98%), 5-hexynoic acid (HexA, 97%, Acros, Geel, Belgium) all-*trans*-retinol (RET, 95%, Acros), poly(ethylene glycol)methyl ether 2-bromoisobutyrate (PEG-Br, M_n_ = 1200 g/mol, Aldrich), sodium azide (NaN_3_, 99%, Acros), *N,N′*-dicyclohexylcarbodiimide (DCC, 99%, Acros), 4-dimethylaminopyridin (DMAP, 99%, Acros), *N,N*-dimethylformamide (DMF, 99%, Chempure, Piekary Śląskie, Poland), l(+)-ascorbic acid (VitC, 99%, Chempure), arbutin (ARB, 95%, Acros), and a 0.1 M sodium phosphate buffer solution (PBS; pH = 7.4, Aldrich) were used as received. All other chemicals were applied without purification.

### 2.2. Synthesis of Alkyne-Functionalized HEMA (2-(Prop-1-En-2-Carbonyloxy)Ethyl Hex-5-Ynate, AlHEMA)

HEMA (3.00 mL, 24.67 mmol) and DCC (5.67 g, 27.48 mmol) were dissolved into a 250-mL round-bottom flask with 50 mL of methylene chloride, yielding a colorless solution. Then, hexynoic acid (2.80 g, 24.97 mmol) was added dropwise to the solution. The reactor was cooled to 0 °C in an ice/water bath, and DMAP (0.1397 g, 1.14 mmol) in methylene chloride (2 mL) was added dropwise. The reaction mixture was stirred for 48 h at room temperature. After that, it was transferred into a separator with methylene chloride and extracted with H_2_O to neutral pH in the aqueous fraction. The organic phase was removed by rotary evaporation. The brown liquid product was dried under vacuum to constant mass. Yield: 61%. ^1^H-NMR (300 MHz, CDCl_3_, ppm): 6.14 and 5.61 (2H, =C**H**_2_), 4.35 (4H, –OC**H**_2_C**H**_2_O–), 2.52 (2H, –OC(=O)C**H**_2_–), 2.28 (2H, –C**H**_2_-C≡CH), 1.99 (1H, –C≡C**H**), 1.95 (3H, –C**H**_3_), 1.81 (2H, –OC(=O)C**H**_2_C**H**_2_–). ^13^C-NMR (300 MHz, DMSO, ppm) (Supplementary Materials Figure S1): 172 (C7, –O**C**(=O)CH_2_–), 166 (C4, –C**C**(=O)O), 136 (C2, CH_2_=**C**–), 126 (C1, **C**H_2_=C–), 83 (C11, –**C**≡CH), 72 (C12, –C≡**C**H), 63 (C5, –O**C**H_2_CH_2_O–), 62 (C6, –OCH_2_**C**H_2_O–), 32 (C8, –OC(=O)**C**H_2_–), 27 (C9, –OC(=O)CH_2_**C**H_2_–), 18 (C10, –**C**H_2_-C≡CH), 17 (C3, –**C**H_3_). Electrospray ionization (ESI) MS (*m*/*z*): calculated for C_12_H_16_O_4_, 224.0; found for [M + Na]^+^, 247.1 (Supplementary Materials Figure S2).

### 2.3. Synthesis of 2-Bromoisobutyrate Derivative of Retinol (3,7-Dimethyl-9-(2,6,6-Trimethylcyclohex-1-En-1-Yl)Nona-2,4,6,8-Tetraen-1-yl 2-Bromo-2-Methylpropanoate, RET-Br)

The RET bioinitiator was prepared with a yield of 95% by esterification with BriBuBr (small excess in relation to molar amount of OH groups) in the presence of TEA according to a previously reported procedure [44]. ^1^H-NMR (600 MHz, DMSO, ppm): 6.50–6.60 (2H, 2* =C**H**–), 6.25–3.35 (1H, =C**H**–), 6.10–6.20 (2H, 2* =C**H**–), 5.60–5.65 (1H, =C**H**–), 4.09–4.12 (2H, –O–C**H**_2_–), 1.98–2.02 (2H, –C**H**_2_– ring), 2.00 (6H, –(C**H**_3_)_2_Br), 1.90–1.92 (3H, –C**H**_3_ ring), 1.75–1.80 (3H, –C**H**_3_ aliphat.), 1.65–1.70 (3H, –C**H**_3_ aliphat.), 1.52–1.60 (2H, –C**H**_2_– ring), 1.40–1.48 (2H, –C**H**_2_– ring), 0.95–1.02 (6H, 2*–C**H**_3_ ring). ^13^C-NMR (300 MHz, DMSO, ppm) (Figure S4, Supplementary Materials): 171 (C18, –O**C**=O), 137.7 (C10, =**C**H–), 137.6 (C11, –**C**(CH_3_)–), 136.3 (C8, C14, –**C**(CH)= ring, =**C**H– ), 131 (C6, C12, =**C**(CH_3_)– ring, =**C**H–), 126.8 (C9, –**C**H=), 126.4 (C15, =**C**H–), 124.5 (C13, –**C**H=), 80 (C16, –**C**H_2_–O), 62 (C19, –**C**–Br), 46 (C3, –**C**H_2_– ring), 34 (C2, –**C**(CH_3_)_2_– ring), 30 (C5, –**C**H_2_– ring), 29 (2*C1, –**C**H_3_ ring), 27 (C20, –(**C**H_3_)_2_Br), 21 (C4, –**C**H_2_– ring), 19 (C7, –**C**H_3_ ring), 9 (2*C17, –**C**H_3_ aliphat.). ESI-MS (*m*/*z*): calculated for C_24_H_35_O_2_Br, 434.9; found for [M + H]^+^, 435.1 (Supplementary Materials Figure S5).

### 2.4. Synthesis of P(AlHEMA-co-MMA) with EiB-Br as Initiator (Example for III) 

MMA (0.24 mL, 2.24 mmol), AlHEMA (1.50 mL, 6.70 mmol), anisole (0.5 mL, 30 vol.% of monomer), and PMDTA (4.66 μL, 0.022 mmol) were placed in a Schlenk flask and then degassed by two freeze–pump–thaw cycles. Then, EiB-Br (3.31 μL, 0.022 mmol) was added and degassed again. After that, CuCl (2.21 mg, 0.022 mmol) was added. The reaction flask was immersed in an oil bath at 60 °C. The polymerization was stopped by exposure to air. Then, the mixture was dissolved in acetone and passed through a neutral alumina column to remove the copper catalyst. The solution was concentrated by rotary evaporation. The polymer was precipitated by dropwise addition of a concentrated solution into diethyl ether. The product was isolated by decantation and dried under vacuum to constant mass.

### 2.5. Synthesis of P(AlHEMA-co-MMA) with RET-Br as Initiator (Example for VI)

RET-Br (14.17 mg, 0.033 mmol), dNdpy (26.70 mg, 0.065 mmol), MMA (0.35 mL, 3.27 mmol), AlHEMA (2.17 g, 9.69 mmol), and anisole (0.25 mL, 10 vol.% of monomer) were placed in a Schlenk flask and then degassed by three freeze–pump–thaw cycles. After that, CuBr (4.60 mg, 0.032 mmol) was added. The reaction flask was immersed in an oil bath at 60 °C. The next steps were performed according to above-described procedure for the synthesis of P(AlHEMA-*co*-MMA) with EiB-Br (Section 2.4).

### 2.6. Synthesis of P(HEMA-co-MMA) (VII–IX)

The series of HEMA-based copolymers with various compositions (HEMA/MMA = 75/25, 50/50, 25/75) were synthesized by ATRP (Table S1, Supplementary Materials) as reported earlier [44]. It was a similar procedure as for AlHEMA, where the copolymerization of HEMA and MMA was performed with the use of RET-Br in the ratio to monomer 1:400 and a CuBr/dNdpy 0.75/1.5 catalyst system in anisole at 60 °C. ^1^H-NMR (300 MHz, DMSO, ppm): 6.00–6.25 (2H_mon_, –CH_2_–), 5.60–5.75 (2H_mon_, –CH_2_–), 4.75–5.00 (1H, –OH–), 4.00–4.20 (2H_mon_, –CH_2_OH), 3.75–4.00 (2H_pol_ –CH_2_OH), 3.75 (2H_mon_, –COCH_2_CH_2_OH), 3.65 (3H_mon_, –OCH_3_), 3.50–3.60 (3H_pol_, –OCH_3_; 2H_pol_, –COCH_2_CH_2_OH), 1.90–2.00 (3H_mon_, –CH_3_; 2H_pol_, –CH_2_– main chain), 0.50–1.50 (3H_pol_, –CH_3_).

### 2.7. Synthesis of Poly(Ethylene Glycol)Methyl Ether 2-Azidoisobutyrate (PEG-N_3_)

PEG-Br (1 g, 0.83 mmol) and NaN_3_ (54.16 mg, 0.83 mmol) were dissolved in a 100-mL round-bottom flask with 20 mL of anhydrous DMF. The reaction mixture was stirred for 24 h at room temperature. After that, it was transferred into a separator with dichloromethane and extracted with NaHCO_3(aq)_. The organic phase was removed by rotary evaporation. The brown liquid product was dried under vacuum to constant mass. Yield: 88%. ^1^H-NMR (300 MHz, DMSO, ppm) (Supplementary Materials Figure S8): 3.50 (n*4H, –[OC**H**_2_C**H**_2_]_n_–), 3.24 (3H, –OC**H**_3_), 1.88 (6H, –C(C**H**_3_)_2_N_3_). ^13^C-NMR (300 MHz, DMSO, ppm) (Supplementary Materials Figure S9): 167 (C4, –O**C**(=O)–), 75 (C2 and C3, –O**C**H_2_**C**H_2_O–), 65 (C1, –O**C**H_3_), 60 (C5, –**C**(CH_3_)_2_N_3_), 41 (C6, –C(**C**H_3_)_2_N_3_).

### 2.8. “Click” Chemistry Azide–Alkyne Reactions (Example for IVc)

Polymer IV (0.37 g, 0.03 mmol containing 0.696 mmol of AlHEMA units) was dissolved into a 100-mL round-bottom flask with 10 mL of DMF. Then, the equimolar amount of PEG-N_3_ (0.90 g, 0.70 mmol) and 2.5-fold molar excess of PMDETA (0.36 mL, 1.74 mmol) were added. The reaction mixture was purged with an inert gas for 20 min. After that, CuBr (0.25 g, 1.74 mmol) was added and the reaction mixture was stirred for 48 h at room temperature without access to light. The mixture was purified from CuBr by means of cationite (Dowex) and concentrated by rotary evaporation. The product was precipitated in diethyl ether and dried under vacuum to constant mass. The reaction efficiency was calculated by integral area of the CH proton in the triazole ring (8.01 ppm, H_M_) and the ≡CH proton from AlHEMA units that were not clicked (1.9–2.0 ppm, H_J_) using the following equation:

$$E_{click}=\frac{H_{M}}{H_{M}+H_{J}}\times 100\%.$$

### 2.9. Incorporation of Active Substance into Polymeric Micelles

The amphiphilic copolymer and active substance were dissolved in methanol with the weight ratio of polymer to bioactive substance = 1:1; then, H_2_O was added dropwise (200 vol.% of the solvent) under gentle stirring. The reaction was continued overnight. After that, the vial with sample was opened to evaporate the organic solvent. The sample was centrifuged to separate the unloaded active substance (4000 rpm for 10 min in room temperature), which was not dissolved. Next, the homogeneous aqueous fraction was collected and lyophilized by freezing. A solution of loaded micelles in MeOH (0.008 mg/mL) was prepared to determine the amount of entrapped substances by ultraviolet–visible light (UV–Vis) spectroscopy, measuring absorbance at λ = 282 nm for ARB and λ = 267 nm for VitC. Drug loading content (DLC) was calculated using the following equation:

$$DLC=\frac{\mathrm{Weight}\;\mathrm{of}\;\mathrm{drug}\;\mathrm{loaded}\;\mathrm{into}\;\mathrm{micelle}}{\mathrm{Weight}\;\mathrm{of}\;\mathrm{total}\;\mathrm{polymer}\;\mathrm{and}\;\mathrm{loaded}\;\mathrm{drug}}\times 100\%.$$

### 2.10. Active Substance Release Studies

The loaded micelles were dissolved in PBS (pH = 7.4, 1.0 mg/mL). The solution was introduced into a dialysis cellulose membrane bag (molecular weight cut-off (MWCO) = 3.5 kDa), which was placed into glass vial with 50 mL of PBS and stirred at 37 °C in a water bath. The buffer solution sample (2.0 mL) was taken from the release medium, at appropriate time intervals, to determine the concentration of released drug by UV–Vis spectroscopy, measuring absorbance at λ = 282 nm for ARB and λ = 267 nm for VitC.

### 2.11. Characterization

^1^H- and ^13^C-NMR spectra were recorded with a UNITY/INOVA (Varian) spectrometer operating at 300 MHz using dimethyl sulfoxide (DMSO) or CDCl_3_ as a solvent and tetramethylsilane (TMS) as an internal standard. The monomer conversion was determined by gas chromatography (GC, Agilent Technologies 6850 Network GC System, Santa Clara, USA). The measurements were carried out in acetone as the solvent. The signals at different retention times corresponded to MMA (2.3 min), HEMA (8.5 min), AlHEMA (10.0 min), and anisole (4.9 min). Mass spectrometry (MS, Xevo G2 QTof, Waters Corporation, Milford, USA) was used to confirm the molecular masses of the modified retinol and functionalized HEMA. Molecular weights (M_n_) and dispersity indices (Đ) were determined by gel permeation chromatography (GPC) equipped with an 1100 Agilent isocratic pump, autosampler, degasser, thermostatic box for columns, and differential refractometer MDS RI Detector. The measurements were carried out in tetrahydrofuran (THF) as the solvent at 30 °C with a flow rate of 0.8 mL/min. The GPC calculations were based on calibration with the use of linear polystyrene standards (580–300,000 g/mol). Fourier-transform infrared spectroscopy (FT-IR) was conducted with Perkin-Elmer Spectrum Two 1000 FT-IR Infrared Spectrometer using attenuated total reflection (ATR). The critical micelle concentration (CMC) was measured by fluorescence spectrophotometry (FL, Hitachi F-2500, Tokyo, Japan), using pyrene as a fluorescence probe. Excitation spectra of pyrene (λ = 390 nm) were recorded at a constant concentration of pyrene (3.0 × 10^−4^ mol/L) and polymer concentrations in the range of 5 × 10^−4^ to 1.0 mg/mL. The intensity ratio (I_336_/I_332_) from the pyrene excitation spectrum vs. logC (where C is the concentration in mg/mL) was plotted, where the cross-over point was estimated as the CMC value. The particle sizes and their distributions, that is, hydrodynamic diameter (D_h_) and polydispersity index (PDI), were measured at 25 °C using dynamic light scattering (DLS, Zetasizer Nano-S90, Malvern Technologies). Each experiment was repeated three times to obtain the average value. The samples taken during the release process were analyzed by ultraviolet–visible light spectroscopy (UV–Vis, Thermo Fisher Scientific Evolution 300) to determine the DLC and the amount of released substance over time. The measurements were carried out in poly(methyl methacrylate) cells. DLC measurements for double-encapsulated systems were carried out using ultra high-performance liquid chromatography–mass spectrometry (UPLC–MS). Analysis was conducted on an ACQUITY UPLC system (Waters) equipped with an ACQUITY photodiode array (PDA) detector and a Waters ACQUITY UPLC^®^BEH C18 column (2.1 × 50 mm, 1.7 mm).

## 3. Results and Discussion

**Figure 1:**
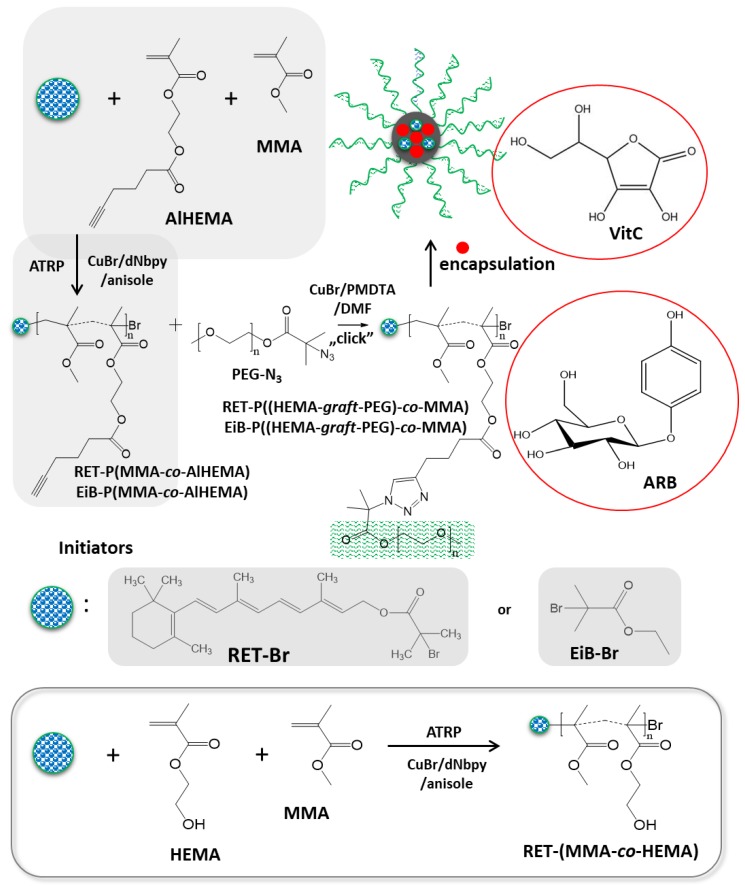
Synthesis of amphiphilic graft copolymers via grafting onto.

A strategy combining the controlled atom transfer radical polymerization (ATRP) and the Cu(I) catalyzed 1,3-dipolar azide-alkyne cycloaddition (CuAAC) was applied in the synthesis of amphiphilic graft copolymers with hydrophilic side chains, e.g., P((HEMA-*graft*-PEG)-*co*-MMA). A few-step procedure, which is presented in Figure 1, included (i) azidation of PEG, (ii) modification of HEMA to alkyne-functionalized monomer (AlHEMA), (iii) its copolymerization with MMA in the presence of different initiators (standard EiB-Br or RET-Br), (iv) the “click” reaction between P(AlHEMA-*co*-MMA) and PEG-N_3_. Varying the content of alkyne groups (recognized as the “clicking” moieties) regulated by MMA units was advantageous to adjust the grafting degree of hydrophilic PEG, whereas a differential hydrophilic–hydrophobic balance influenced the behavior in aqueous solution. The self-assembly and delivery of the selected bioactive substances by the grafted copolymers were compared with the systems of linear amphiphilic copolymers of HEMA and MMA (combined with various proportions).

**Figure 2:**
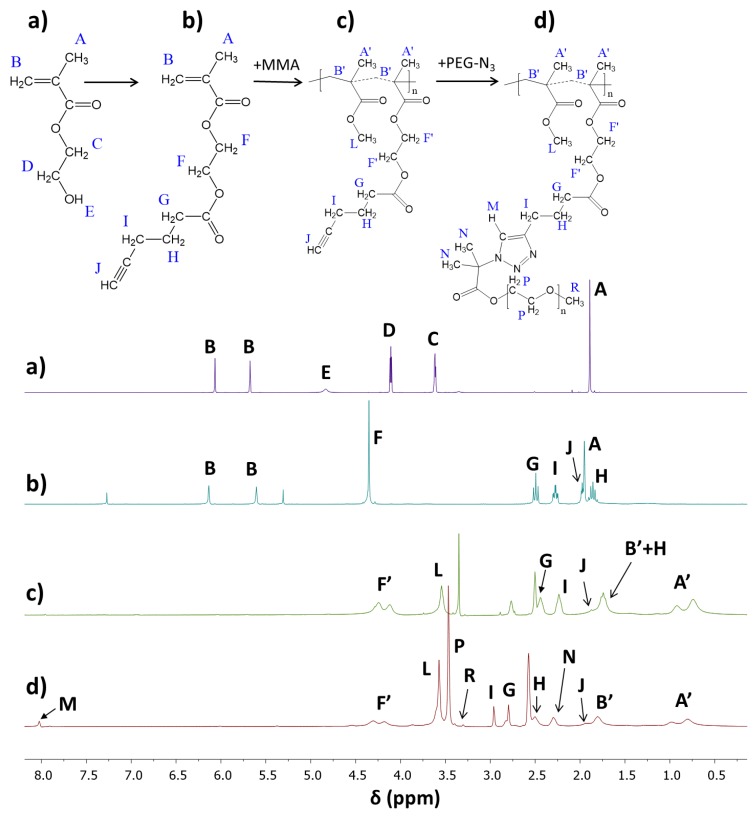
^1^H-NMR spectra of (**a**) HEMA, and (**b**) after its modification AlHEMA, (**c**) P(MMA-*co*-AlHEMA) V, (**d**) P((AlHEMA-*graft*-PEG)-*co*-MMA) Vc.

The alkyne derivative of HEMA (AlHEMA) was obtained by the coupling reaction via esterification between the OH group of HEMA and hexynoic acid. The structure of the resultant AlHEMA was confirmed by ^1^H-NMR, showing the shifted methylenoxy signals **C** and **D** at 3.61 and 4.11 ppm (Figure 2a) as the signal **F** at 4.35 ppm (Figure 2b) due to neighborhood changes following the formation of an ester group and introduction of the hexynoic moiety (Figure 2b: 2H, **H:** 1.8–1.9 ppm; 1H, **J:** 2.0 ppm; 2H, **I:** 2.2–2.4 ppm, and 2H, **G:** 2.4–2.6 ppm).

Similarly, the retinol was modified by esterification to an ATRP bioinitiator with bromoester functionality. In the ^1^H-NMR spectrum, the signal of the hydroxyl group (Supplementary Materials Figure S3a; **p:** 4.7 ppm) disappeared after modification, whereas the signal of the methylene group in –C**H_2_**OH was shifted (Supplementary Materials Figure S3a,b; **o:** from 4.1 to 4.8 ppm) due to the presence of the ester group. The successful modification was also confirmed by the ^13^C-NMR spectra (Supplementary Materials Figure S4) containing the signals, which corresponded to >C=O in the introduced ester group (**C18:** 170 ppm), carbon associated with the ester group (**C16:** 80 ppm), and tertiary carbon bonded to bromine (**C19:** 63 ppm). The lack of a broad band in the region of 3100–3600 cm^−1^ corresponding to ν(O–H) stretching in the FT-IR spectra of esterified RET, the presence of the additional peak at 1260 cm^−1^ from the stretching vibration of C–O, the strong peak at 1730 cm^−1^ from the stretching vibration of C=O groups, and the strong peak at 800 cm^−1^ from the stretching vibration of C–Br were evidence for the newly created bromoester group (Supplementary Materials Figure S6).

**Figure 3:**
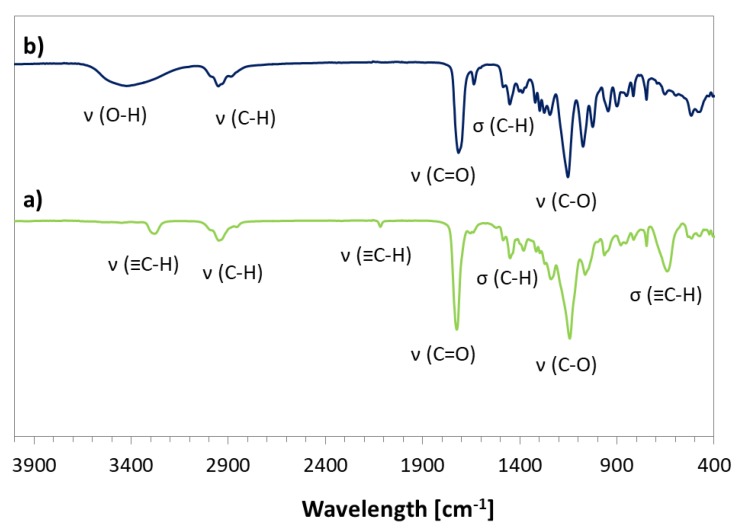
Fourier-transform infrared (FT-IR) spectra for copolymers (**a**) P(AlHEMA-*co*-MMA) III and (**b**) P(HEMA-*co*-MMA) IX.

The prepared bromoester-functionalized retinol (RET-Br) and commercially available standard ATRP initiator, e.g., EiB-Br, were applied in the copolymerization of AlHEMA in the presence of a CuBr/dNbpy or CuCl/PMDTA catalyst system in anisole at 60 °C (Table 1). The range of initiators was broadened by RET-Br to develop the biocompatibility of polymers, but it also motivated characterizing its influence on the reaction rate and properties of the copolymers, including the structural parameters (i.e., degree of polymerization (DP), dispersity index (Ɖ)) in comparison to that obtained with the use of EiB-Br. The introduction of alkyne groups into monomer prior to the polymerization reaction guaranteed that the alkyne functionality was contained in all HEMA units incorporated into the polymer chain, whereas, in the case of the post-polymerization modification of HEMA-based polymers, it was strongly dependent on the esterification efficiency. The used strategy of pre-polymerization modification was beneficial for the adjustment of a certain number of alkyne groups in the copolymer by the initial proportions of AlHEMA/MMA comonomers (25/75, 50/50, 75/25). The alkyne moieties in copolymers were observed on the FT-IR spectra (Figure 3b) as peaks at 550–700 cm^−1^ and 3300 cm^−1^ from the bending and stretching vibrations of ≡C–H, respectively. It was in contrast to the hydroxy-functionalized polymers (Figure 3a), which revealed a broad band in the region of higher values of wavelengths corresponding to ν(O–H) stretching in HEMA units.

The conversion of AlHEMA was calculated from the ^1^H-NMR spectrum for the reaction mixture (Supplementary Materials Figure S7) using protons in methylene groups via integration of signals corresponding to the monomer (CH_2_=, **B:** 5.6 and 6.2 ppm) and polymer (–COO–CH_2_–, **F’:** 4.1–4.2 ppm). The determination of MMA conversion was based on protons in the methoxy group via integration of signals corresponding to the monomer (**L:** 3.75 ppm) and polymer (**L’:** 3.6 ppm). However, for further calculations of DP and molecular weight (M_n_), the conversion by GC was selected due to the very good separation of signals, which was not always possible in the case of ^1^H-NMR analysis. Comparable values of monomer conversions (AlHEMA vs. MMA) allowed concluding the formation of statistical copolymers. The detailed data are summarized in Table 1, whereas the representative ^1^H-NMR spectrum of the purified copolymer P(AlHEMA-*co*-MMA) is presented in Figure 2c.

**Figure 4:**
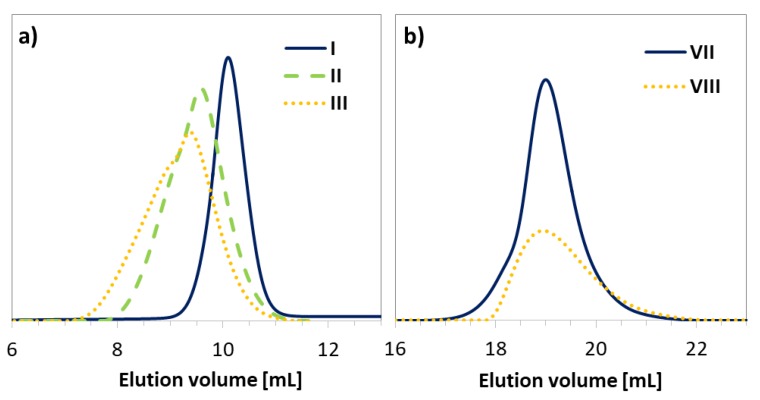
GPC traces for copolymers (**a**) P(AlHEMA-*co*-MMA) and (**b**) P(HEMA-*co*-MMA).

Dependent on the initiator (EiB-Br vs. RET-Br), the polymerization progress was characterized with different rates and total monomer conversions. The use of a standard EiB-Br initiator resulted in higher conversions within a shorter time compared to RET-Br (45% within 4.5 h vs. 25% within 24 h). However, the biological and non-toxic nature of the retinol starting unit can be beneficial for the improvement of skin treatment, whereas the resulting conversions are sufficient to obtain copolymers with the desired properties. In the case of HEMA polymerization initiated by RET-Br (Supplementary Materials Table S1), the reactions were significantly faster than the series of AlHEMA/RET-Br giving similar conversions in a shorter time (IV vs. VII = 23/22 vs. 18/18; VI vs. IX = 26/30 vs. 26/32 at 24 h vs. 0.5–4.5 h), which is rational due to more polar HEMA-based systems. The obtained copolymers were characterized by moderate dispersity indices usually exceeding 1.5, but their GPC traces were monomodal and symmetrical, showing some broadness and discrepancy with the increase in the content of alkyne or hydroxyl moieties (Figure 4). 

### Click Reactions

A commercially available low-molecular-weight bromoester monofunctionalized PEG was converted by the substitution reaction of the bromine atom with an azide group. In the ^1^H-NMR spectrum, the signals coming from methyl groups adjacent to –Br or –N_3_ were placed in the same range of chemical shifts giving multiplets at 1.82–1.92 ppm or a broad signal at 1.86–1.90 ppm, respectively (Supplementary Materials Figure S8). Because of the non-synonymous approval, the final identification of the end bromoester group in PEG was provided by ^13^C-NMR analysis, which confirmed the achievement of the azidation reaction. The signal derived from carbon of the carboxyl group was shifted toward lower chemical shifts (**C4**: 172 ppm to 168 ppm) due to the impact of a new group attached to the adjacent carbon (**C5**: 63 ppm to 60 ppm), whereas the signals of the methylene groups moved toward higher chemical shifts (**C6:** 37 ppm to 40 ppm) (Supplementary Materials Figure S9). The molecular weight of the PEG derivative determined by GPC was consistent with the weight calculated from ^1^H-NMR, giving similar values of M_n_ for PEG-N_3_ (1380 vs. 1300 g/mol by NMR vs. GPC, respectively) (Supplementary Materials Figure S10). Additionally, the impurities contained in the commercial PEG were removed by purification of the modified PEG, which resulted in the reduction of the dispersity index determined by GPC. 

**Figure 5:**
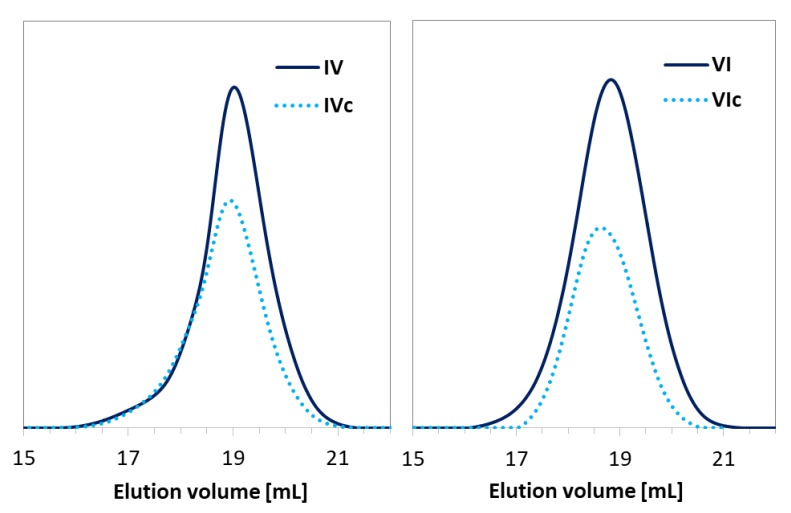
GPC traces for copolymers before (IV, VI) and after “click” reaction (IVc, VIc).

The hydrophilic PEG-N_3_ chains were grafted onto the multifunctional P(MMA-*co*-AlHEMA) by a Huisgen “click” chemistry CuAAC reaction between azide and alkyne moieties catalyzed by CuBr/PMDTA in DMF with the formation of 1,4-substituted triazole rings (Figure 1). It was found that dispersity indices of the resulted amphiphilic graft copolymers were reduced in comparison to their backbones before the “click” reaction (Table 2, Figure 5). Generally, the discrepancy of M_n,GPC_ from M_n,NMR_ was more significant than for the linear copolymers because of the lower hydrodynamic volumes of nonlinear macromolecules, which were applied as standards for the calibration in GPC. Additionally, higher-molecular-weight distribution can influence such a deviation. 

The presence of a triazole proton at 8.01 ppm (H_M_) and three groups of signals from PEG (methoxy group at 3.29 ppm (H_R_), methylene groups at 3.45 ppm (H_P_), and methyl groups at 2.30 ppm (H_N_)) in the ^1^H-NMR spectrum validated the success of the “click” reaction (Figure 2d). In the series Ic–IIIc, independently of the amount of alkyne groups, the “click” efficiency (E_click_) reached the level of 30% (Table 2). It was related to grafting of 11–34 side PEG chains (synonymous with the number of triazole rings (n_triazole_)), which was adjusted by the content of alkyne groups per backbone (F_AlHEMA_). Various grafting degrees (DGs) also corresponded to an almost equimolar content of the introduced hydrophilic fraction (47–57 wt.%). Significant differences were observed for the RET series characterized with the lowest (IVc) and the highest (VIc) “click” efficiencies (21% vs 64%). The retinol-initiated copolymers IV and VI used for the “click” reaction were varied from the analogical I and III not only by the starting group (RET vs. EiB), but also with their structural parameters (F_AlHEMA_ as the result of DP_AlHEMA_ in relation to the chain length). A two-fold lower number of “clickable” alkyne moieties in the copolymer IV than in sample I (DP_AlHEMA_ = 79 vs. 136) at a similar content of alkyne groups (F_AlHEMA_ ~72%) appeared to be more effective in the PEG grafting. 

**Figure 6:**
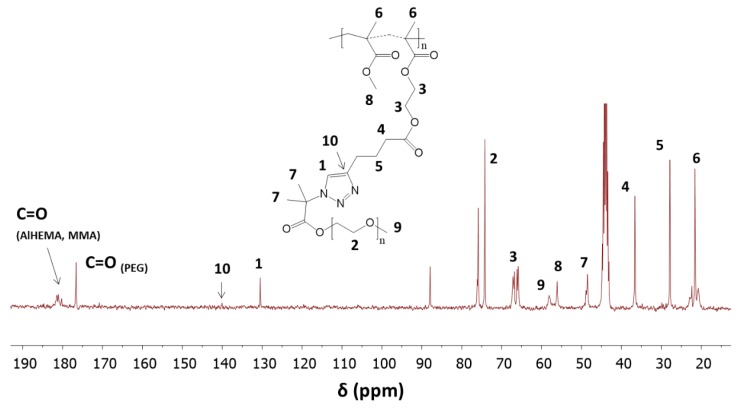
^13^C-NMR of graft copolymer P((HEMA-*graft*-PEG)-*co*-MMA) by “click” reaction.

**Figure 7:**
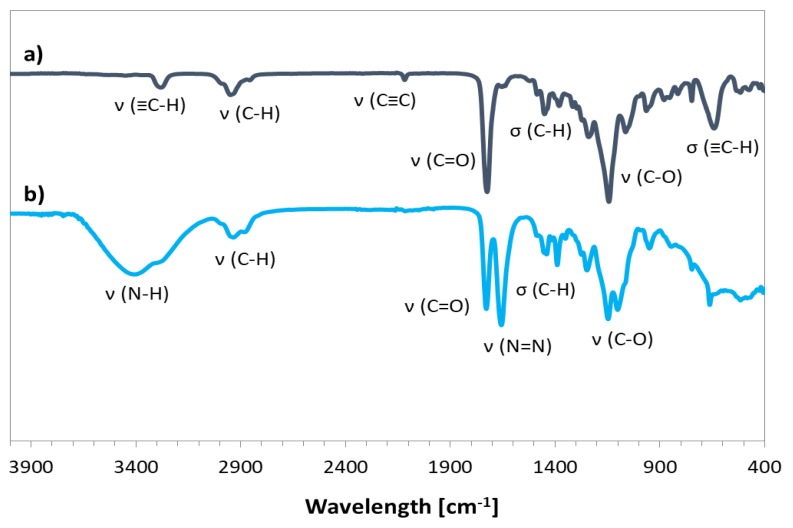
FT-IR spectra for P(AlHEMA-*co*-MMA) (**a**) and P((HEMA-*graft*-PEG)-*co*-MMA) (**b**).

Chemical structures of the graft copolymers were also confirmed using ^13^C-NMR and FT-IR spectroscopies. The presence of signals from carbons of the triazole ring (**C_1_**, 130 ppm; **C_10_**, 140 ppm) and the carbon signal of the carboxyl group at the PEG chain (176 ppm) indicated the success of the “click” reaction (Figure 6). In the FT-IR spectrum, the broad band in region of 3000–3600 cm^−1^ corresponding to ν(N–H) stretching and the strong peak at 1650 cm^−1^ from ν(N=N) in the triazole ring were observed (Figure 7).

The self-assembling behaviors of the graft copolymers were investigated by determination of the critical micelle concentration (CMC, Table 2). CMC values of copolymers were measured by a standard procedure using the emission spectra of pyrene to form the plot of I_336_/I_332_ vs. the logarithm of the copolymer concentration (Supplementary Materials Figure S11). It was noted that the CMC value decreased with the increase in hydrophobic fraction in the range of series (Ic–IIIc, and IVc vs. VIc), which is the general relationship in the self-assembly process. The highest CMC value was observed for the copolymer with a predominated content of hydrophilic fraction (VIc), which was generated by the highest degree of PEG grafting, demonstrating the lowest ability for micellization compared to the other systems. Copolymers Ic–IIIc with equimolar hydrophilic/hydrophobic fractions (47–57 wt.%) showed similar CMC values in the range of 0.01–0.02 mg/mL. Previously investigated linear hydroxyl-functionalized copolymers of P(HEMA-*co*-MMA) self-assembled at lower concentrations (0.002–0.04 mg/mL, Supplementary Materials Table S1) [44] compared to grafted copolymers with analogical backbones, which confirmed that the presence of PEG side chains improved the system solubility.

Satisfactory results obtained in the case of encapsulation of cosmetic substances (ferulic acid, VitC) into micelles of the linear HEMA-based copolymers [44] encouraged us to investigate the self-assembling graft polymers in the presence of ARB or VitC. These model bioactive substances are well-known components in cosmetics due to their antioxidant and skin-lightening activities. ARB prevents the formation of melanin-avoiding skin diseases, such as melanoma, and it is used as replacement of hydroquinone, whereas VitC inhibits the influence of free radicals, stimulates collagen synthesis, and is used with α-tocopherol for a synergistic effect. The efficiency of the single-drug encapsulation (performed in the ratio of polymer to drug 1:1) was verified by drug loading content (DLC, Table 3), which was determined by the use of UV–Vis spectra. ARB was encapsulated in larger amounts than VitC by grafted copolymers (~90% vs. ~15%), which was in contrast to the linear copolymers (~50–75% vs. ~80%). There was no effect of the grafting degree (Ic–IIIc, IVc vs. VIc) on the DLC, although the RET-based series was more efficient in ARB encapsulation than the EiB one (~90% vs. ~55%). The loading results allowed concluding that the structure of copolymer, including topology and localization of hydrophilic moieties in the polymer, and the nature of active substance are crucial factors when designing the encapsulated systems with an optimized amount of the cosmetic substance. The encapsulation of two active substances (VitC and ARB) at the same time was also attempted. The DLC calculations were performed by UHPLC–MS measurements because the bands of VitC and ARB overlapped in the UV–Vis spectrum. However, the results indicated that only ARB was encapsulated. This means that these systems are not sufficient for the dual delivery of these two specific active substances, whereas double encapsulation may be successful for other bioactive pairs.

**Figure 8:**
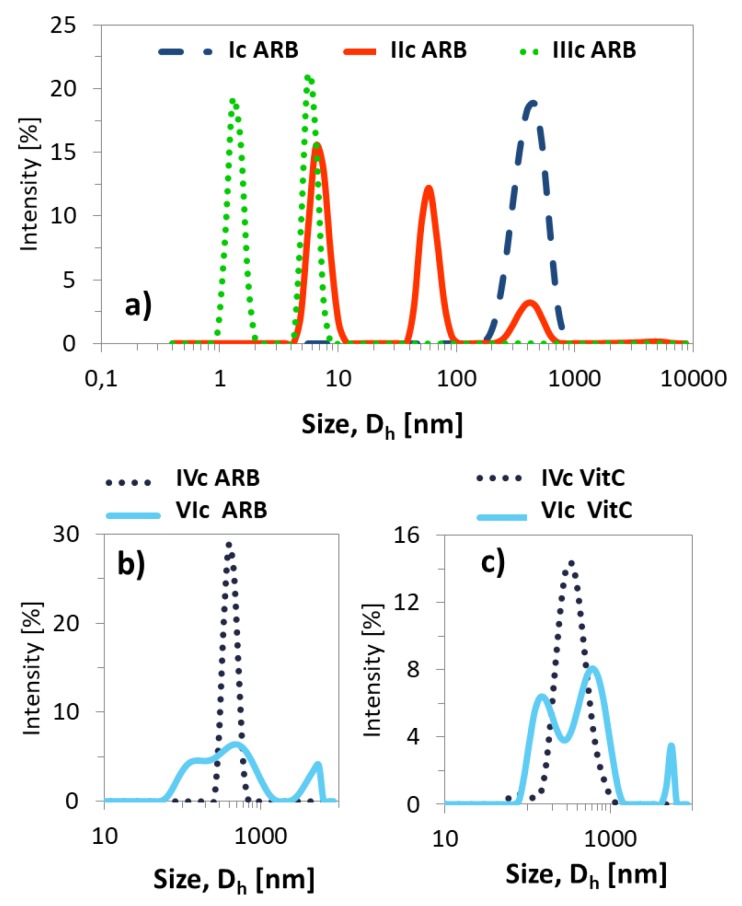
Size distribution plots by intensity for ARB- (**a**,**b**) or VitC (**c**) loaded polymer micelles in PBS at 25 °C.

**Figure 9:**
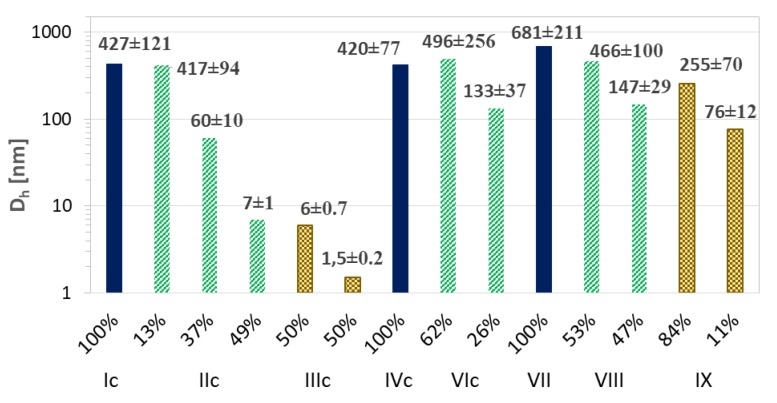
Particle size distribution data for ARB-loaded micellar systems based on intensity calculation method.

The resulting ARB or VitC encapsulated particles were analyzed by DLS in PBS solution (Table 3). Micelles containing ARB obtained from the grafted copolymers (Ic–VIc) were smaller compared to their linear counterparts (VII–IX). There was no significant effect for samples Ic and IVc differing with the initial unit (EiB vs. RET) and similar DG, which yielded one fraction of micelles; however, at a higher content of side chains, more fractions of the superstructures with different hydrodynamic diameters were observed (Figure 8). A few generations of loaded particles may indicate the presence of unimers, as well as the formation of micelles and aggregates (Figure 9, Supplementary Materials Figure S12). Similar correlations were detected for the systems based on the linear amphiphilic analogs, although the size differences were more spectacular.

**Figure 10:**
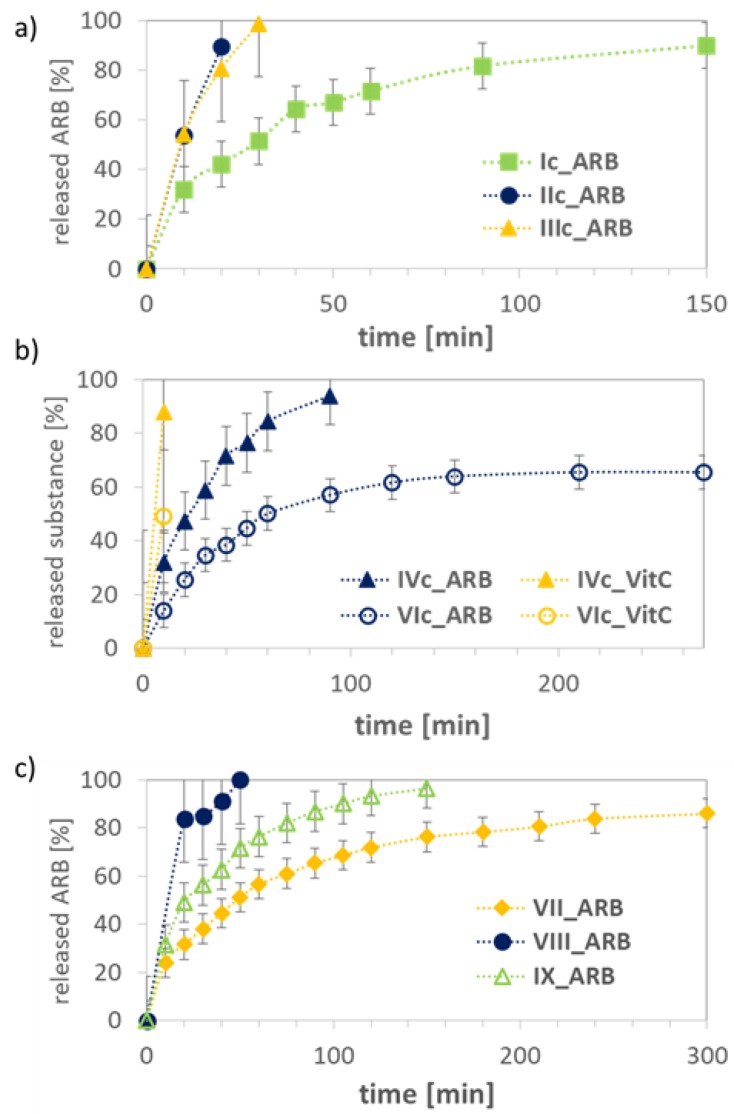
Release profiles for micellar systems formed by graft copolymers (**a**,**b**) and linear copolymers (**c**).

The release experiments were carried out in PBS at pH 7.4 for the ARB- or VitC-loaded systems, which demonstrated various release rates of bioactive substance dependent on the hydrophilic/hydrophobic balance and topology of carriers, including the length of the main chain/backbone (Table 3, Figure 10). The kinetic profiles showed a tendency of increasing rate of ARB release with the decrease in hydrophilic content for series of graft copolymers with backbone DPs ~100 (VIc: (F_hydrophilic_ = 76%) ARB release 35%, Ic: (47%) 50%, and IVc: (35%) 60% within 30 min). However, this correlation was not valid for IIc and IIIc, showing significantly faster drug release than system Ic with a comparable amount of hydrophilic fraction (~100% vs. 50% within 30 min); however, contrary to the other graft copolymers, they contained two-fold longer backbones contributing to micelle core formation. For almost all systems based on the graft copolymers, the release of ARB was completed at ~90–100% with the exception of VIc with the largest grafting degree and the highest hydrophilic content. This discrepancy can be explained by the formation of micelles with a thicker outer layer of hydrophilic PEG, which decelerated the drug release. A similar effect was observed for VitC, although its release was faster than for ARB (for IVc 88% VitC vs. 32% ARB and for VIc 49% VitC vs. 14% ARB within 10 min, Figure 10b). In the case of linear hydroxyl-functionalized copolymers, the release of ARB ranged from 86–100% and it was strongly dependent on the chain length, showing the fastest release by the longest chain with an equimolar content of hydrophilic fraction (VIII DP = 136) and the slowest release by the shortest chains with a predominated hydrophobic fraction (VII DP = 73) (Figure 10c). The randomly distributed hydrophilic HEMA units along the polymeric chains were responsible for different correlations between release rate and hydrophilic content from that observed for the graft polymers containing side PEG segments. Surprisingly, both ARB and VitC were released faster from the graft copolymer systems than from their linear analogues. However, a short release time is beneficial from the point of view of applying the designed systems in cosmetic products due to the short time of application on the skin.

The release kinetics of ARB was also described by fitting to mathematical models, which were represented as semilogarithmic plots of remaining drug vs. time according to the first-order equation, and plots of the cumulative amount of released drug vs. square root of time according to the Higuchi model. Both types of plots (Supplementary Materials Figures S13 and S14) demonstrated good correlation coefficients, that is, 0.89–0.99 for Ic–VIc (graft copolymers), 0.93–0.99 for VII–IX (linear copolymers), 0.85–0.99 for Ic–VIc, and 0.87–0.96 for VII–IX. These results confirmed the concentration-dependent and diffusion-controlled mechanism.

## 4. Conclusions

Pre-polymerization modification was applied to obtain the alkyl-functionalized monomer originating from HEMA, which was used for copolymerization via ATRP with bromoester initiators, including the modified retinol. The pre-polymerization strategy provided much better control of the number of alkyne groups in the copolymer, which could be modified into the amphiphilic graft copolymer via a “click” reaction between the alkyne functionality in the HEMA-based copolymer and the azide-functionalized PEG. The self-assembling behavior in aqueous solution at room temperature was employed to encapsulate ARB or VitC into micelles with a relatively high efficiency for almost all systems (DLC > 50%). In vitro release was carried out indicating the maximum amount of released ARB after 20 min (up to 5 h) and VitC after 10 min. With respect to both encapsulation and release studies, the PEG graft copolymers seem to be good candidates for potential delivery applications. The micellar systems with a short release time (up to 30 min) can be effective in face masks, whereas the other ones delivering bioactive substances over a longer time are perfect for cream applications. All these systems need to be tested for toxicity and diffusion through artificial skin to verify their application in cosmetology (masks, under-eye patches, and wraps).

## Figures and Tables

**Table 1:** Data for synthesis of AlHEMA/MMA copolymers by ATRP.

	M_1_/M_2_	Time (h)	Conversion (%)				DP_n,GC_	M_n,GC_ (g/mol)	M_n_^a^ (g/mol)	D ^a^
			NMR		GC					
			M_1_	M_2_	M_1_	M_2_				
I	25/75	4.0	16	28	34	27	115	15,900	21,400	1.36
II	50/50	4.5	48	50	47	52	198	31,600	33,600	2.06
III	75/25	4.5	46	45	42	47	173	33,100	35,500	1.83
IV	25/75	24	33	29	23	22	89	12,200	25,400	1.68
V	50/50	24	27	15	18	17	71	12,000	17,800	1.72
VI	75/25	24	32	24	26	30	109	21,000	30,400	1.65

I: [AlHEMA+MMA]_0_/[EiB-Br]_0_/[CuBr]_0_/[dNdpy]_0_ = 400/1/0.75/1.5; II–III: [AlHEMA+MMA]_0_/[EiB-Br]_0_/[CuCl]_0_/[PMDTA]_0_ = 400/1/1/1; IV–VI: [AlHEMA+MMA]_0_/[RET-Br]_0_/[CuBr]_0_/[dNdpy]_0_ = 400/1/1/2; anisole 10 vol.% of mon., 60 °C; ^a^ determined by GPC in THF with polystyrene standards.

**Table 2:** Data for the synthesis of graft copolymers P((HEMA-*graft*-PEG)-*co*-MMA).

	DP_AlHEMA_	F_AlHEMA_ (%)	E_click_ (%)	n_triazole_	DG (%)	F_hydrophilic_ (wt%)	M_n,NMR_ (g/mol)	M_n,GPC_^a^ (g/mol)	D ^a^	CMC (mg/mL)
Ic	34	30	33	11	10	47	28,900	58,600	3.40	0.0159
IIc	94	47	31	29	15	54	69,300	24,100	1.34	0.0169
IIIc	126	73	27	34	20	57	77,300	36,500	1.50	0.0229
IVc	23	26	21	5	6	35	17,200	29,000	1.62	0.0836
VIc	79	72	64	51	47	76	75,000	35,900	1.42	0.4283

^a^ Determined by GPC in THF with polystyrene standards; DP_AlHEMA_—polymerization degree of AlHEMA; F_AlHEMA_ and F_hydrophilic_—content of AlHEMA and hydrophilic fraction in the copolymer, respectively; E_click_—efficiency of “click” reaction; n_triazole_—number of triazole moieties in the copolymer; DG—grafting degree.

**Table 3:** Characteristics of encapsulated particles and release of active substances.

	D_h_^a^ (nm)		PDI		DLC (%)		Maximum Amount of Released Drug (%)/Time (h)	
	ARB	VitC	ARB	VitC	ARB	VitC	ARB	VitC
Ic	427	-	0.508 ± 0.028	-	64	-	90/2.5	-
IIc	80 ^b^	-	0.717 ± 0.018	-	49	-	90/0.3	-
IIIc	7 ^b^	-	0.983 ± 0.016	-	56	-	99/0.5	-
IVc	420	369	0.241 ± 0.046	0.200 ± 0.063	99	16	94/1.5	88/0.17
VIc	310 ^b^	377 ^b^	0.634 ± 0.007	0.621 ± 0.085	87	13	65/4.5	49/0.17
VII	681	834 ^c^	0.093 ± 0.053	0.261 ± 0.081	55	87 ^c^	86/5.0	62/1.0 ^c^
VIII	316 ^b^	250 ^c^	0.904 ± 0.002	0.281 ± 0.060	48	78 ^c^	100/0.8	48/1.0 ^c^
IX	222 ^b^	-	0.691 ± 0.086	-	75	-	96/2.5	-

^a^ Particle size distribution data based on intensity calculation method; ^b^ non-dominated fraction—the averaged value for the major fractions above 80%; ^c^ data presented in Reference [44]; Ic–VIc: graft copolymers; VII–IX: linear copolymers.

## Supplementary Materials

The following are available online at https://www.mdpi.com/1999-4923/11/8/378/s1. Data for synthesis of P(HEMA-*co*-MMA) copolymers; ^1^H-NMR spectra of retinol before and after esterification, PEG-Br and PEG-N_3_, reaction mixture of copolymerization AlHEMA/MMA; ^13^C-NMR spectra of AlHEMA, RET-Br, PEG-Br, and PEG-N_3_; ESI-MS spectra of AlHEMA and RET-Br; FT-IR spectra and GPC traces for PEG-Br and PEG-N_3_; CMC plots; particle size distribution for VitC-loaded systems; kinetic plots.
