# Supplementary material for: Retinol-Containing Graft Copolymers for Delivery of Skin-Curing Agents
Source: Pharmaceutics. 2019 Aug 2;11(8):378. doi: 10.3390/pharmaceutics11080378 (PMC6722771; doi:10.3390/pharmaceutics11080378)
Supplement: Supplementary file 1 [file pharmaceutics-11-00378-s001.pdf]

pharmaceutics

MDPI

Article

Retinol-Containing Graft Copolymers for Delivery of Skin-Curing Agents

Justyna Odrobińska [1], Katarzyna Niesyto [1], Karol Erfurt [2], Agnieszka Siewniak [2], Anna Mielańczyk [1] and Dorota Neugebauer [1,*]

Table S1. Data for synthesis of P(HEMA-*co*-MMA) copolymers by ATRP[a].

	M_1/M_2	Time [h]	Conversion (%) NMR M_1	Conversion (%) NMR M_2	Conversion (%) GC M_1	Conversion (%) GC M_2	$DP_{n,GC}$	$M_{n,GC}$ (g/mol)	GPC[b] M_n (g/mol)	GPC[b] Đ	CMC (mg/mL)
**VII**	**25/75**	**4.5**	**21**	**16**	18	18	73	8 200	24 300	1.42	0.0016/0.0057
**VIII**	50/50	2.7	32	24	31	37	136	15 900	17 200	1.70	0.0182
**IX**	75/25	0.25	28	41	26	32	122	13 700	nd	nd	0.0433

$[HEMA+MMA]_0/[RET\text{-}Br]_0/[CuBr]_0/[dNdpy]_0$ = 400/1/0.75/1.5; anisole 10 vol. % of mon., 60°C; [a]data presented in ref. [44]; [b] THF; nd – not determined;

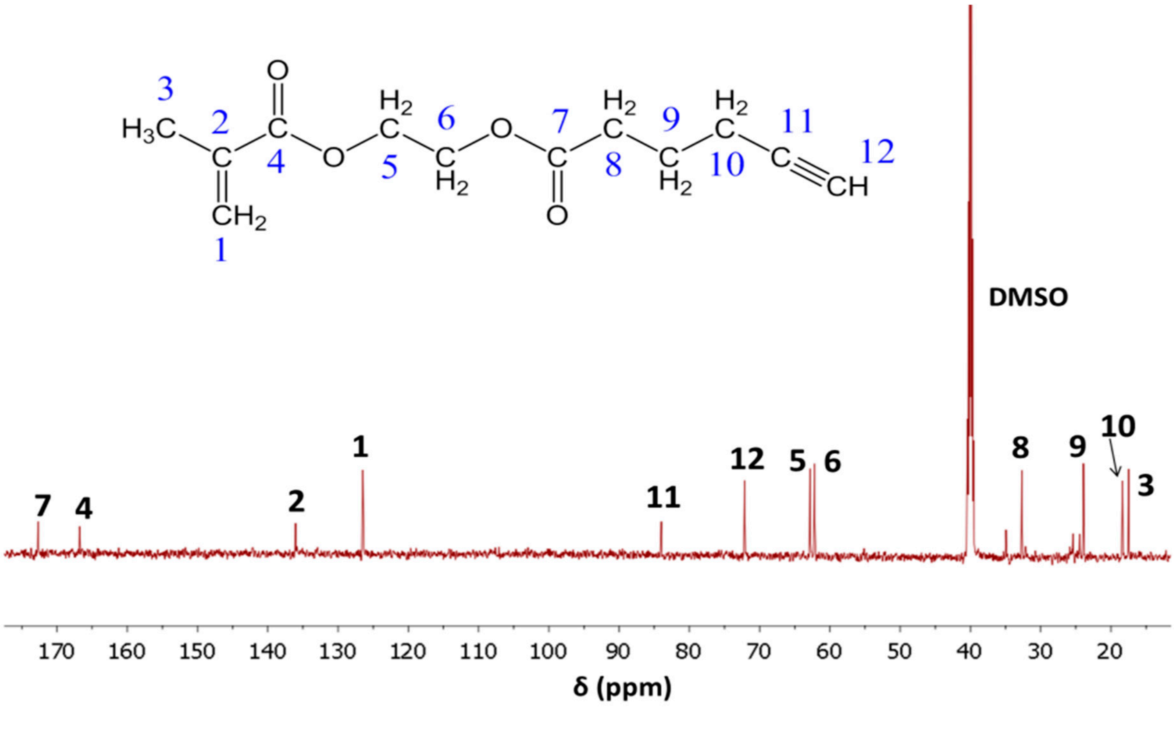

Figure S1. ^{13}C NMR spectra of AlHEMA.

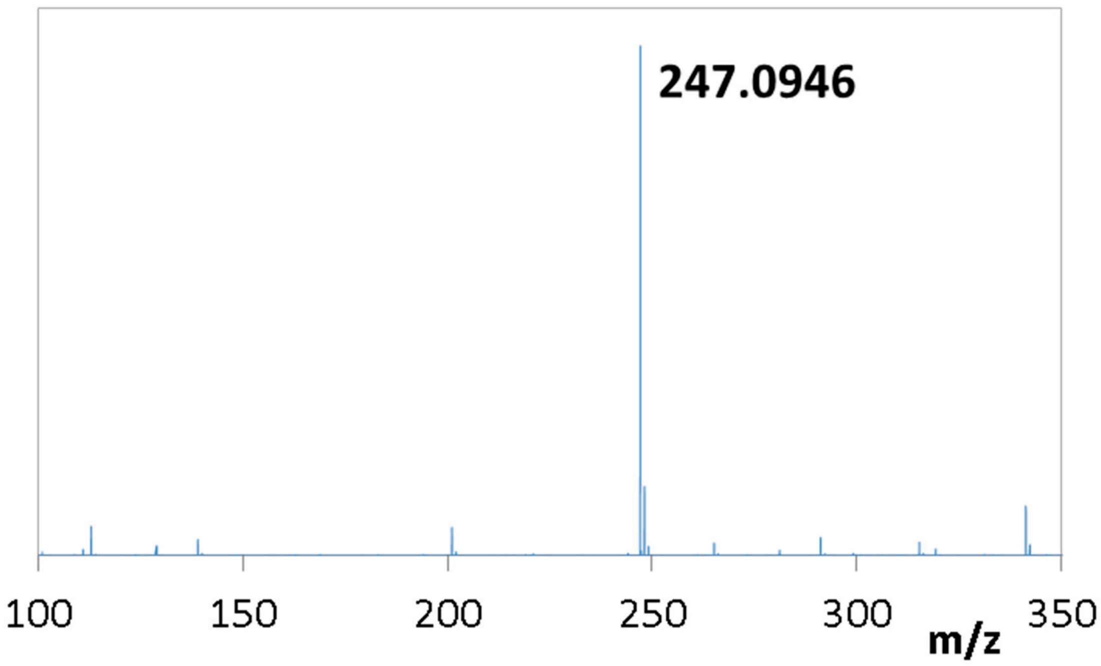

Figure S2. ESI-MS spectra of AlHEMA.

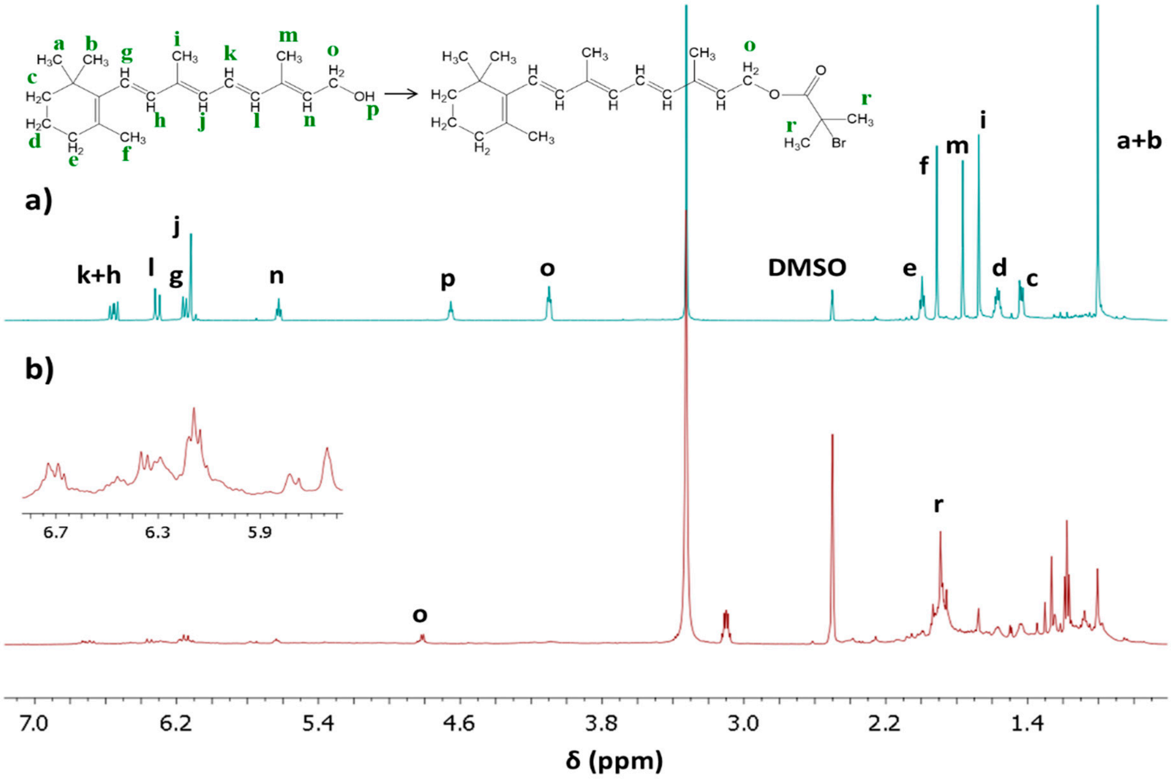

Figure S3. ^{1}H NMR spectra in DMSO of a) RET, and b) RET-Br.

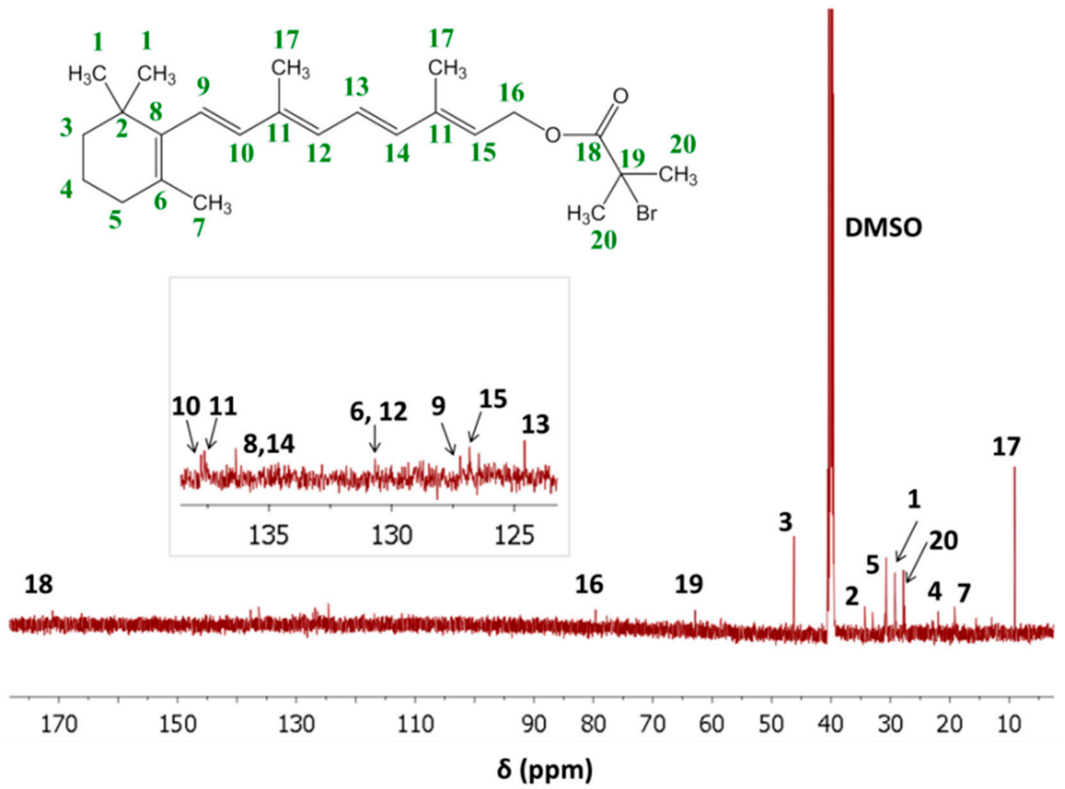

Figure S4. ^{13}C NMR spectra of RET-Br.

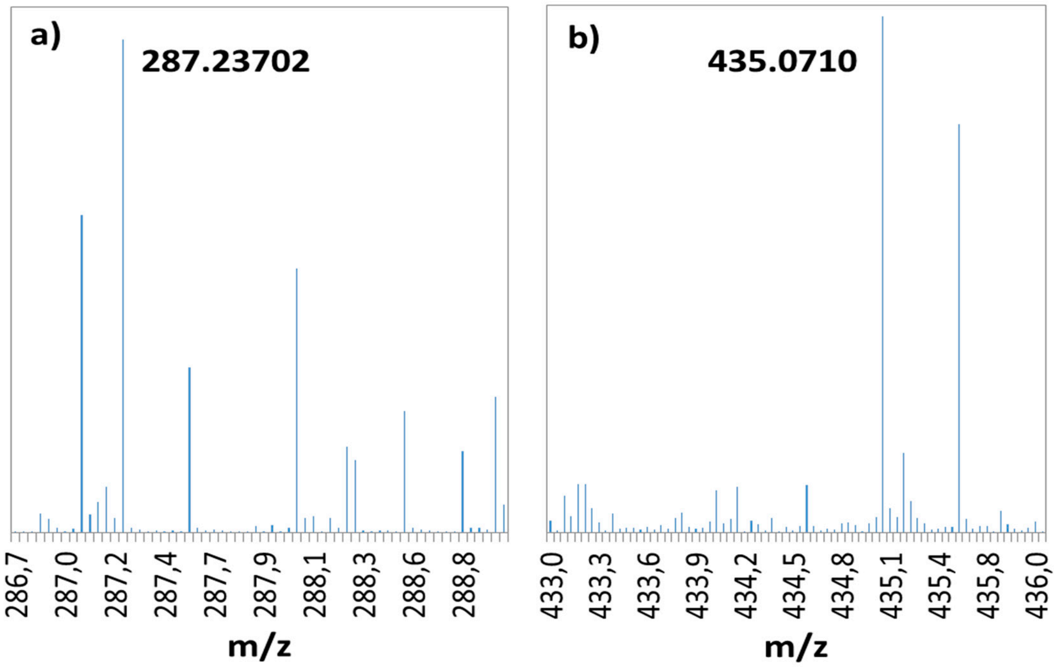

Figure S5. ESI-MS spectra of a) RET, b) RET-Br. calculated for RET: $C_{20}H_{30}O$ 286.0, found for $[M+H]^+$ 287.2.

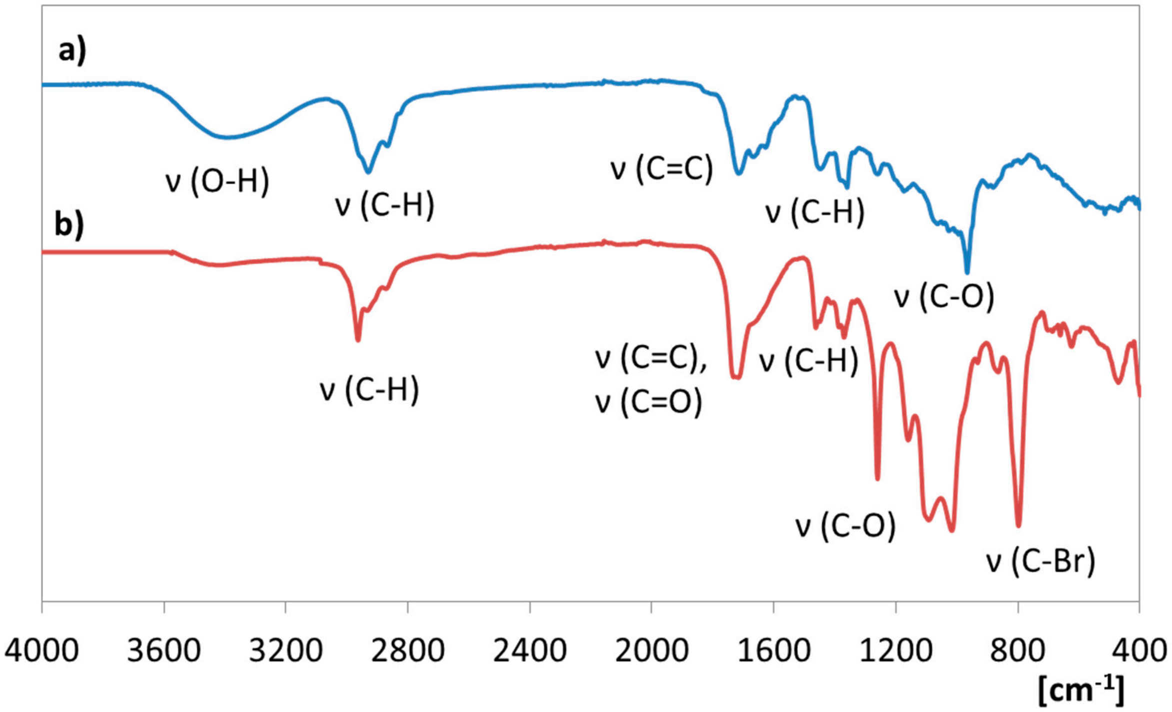

Figure S6. FT-IR spectra of a) RET, b) RET-Br.

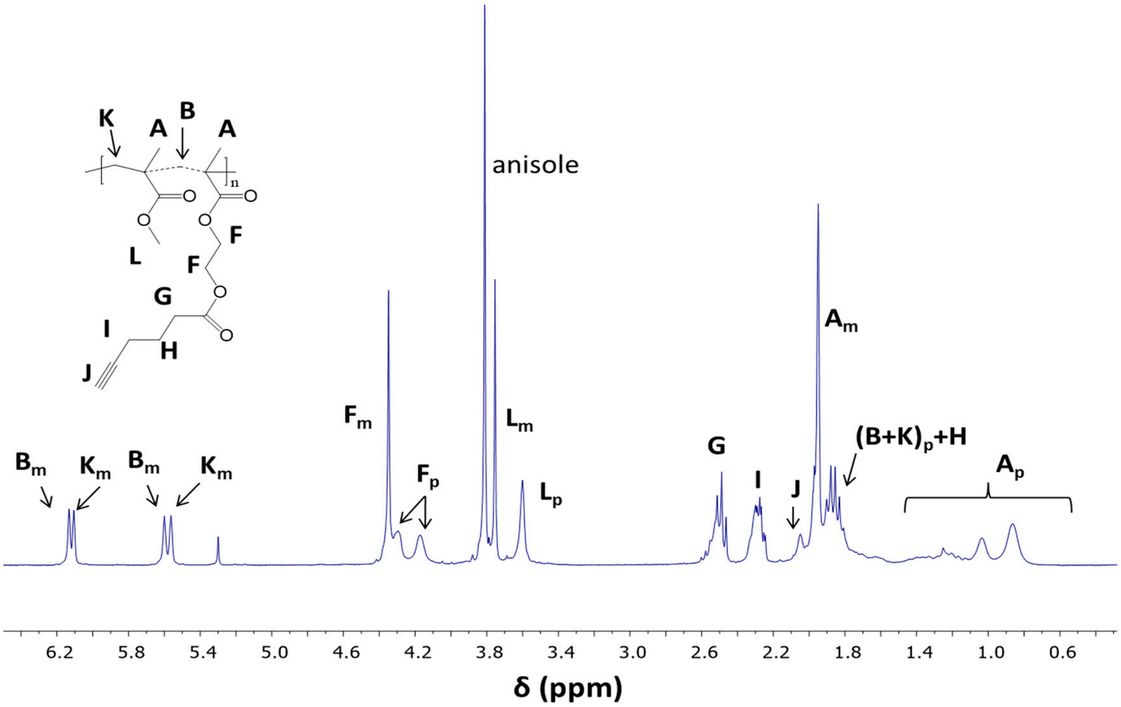

Figure S7. ^{1}H NMR spectra ($CDCl_3$) of the sample taken from the reaction mixture for EiB-Br initiated copolymerization of AlHEMA/MMA: 50/50 (II), where signals with indices *m* and *p* are related to monomer and polymer, respectively.

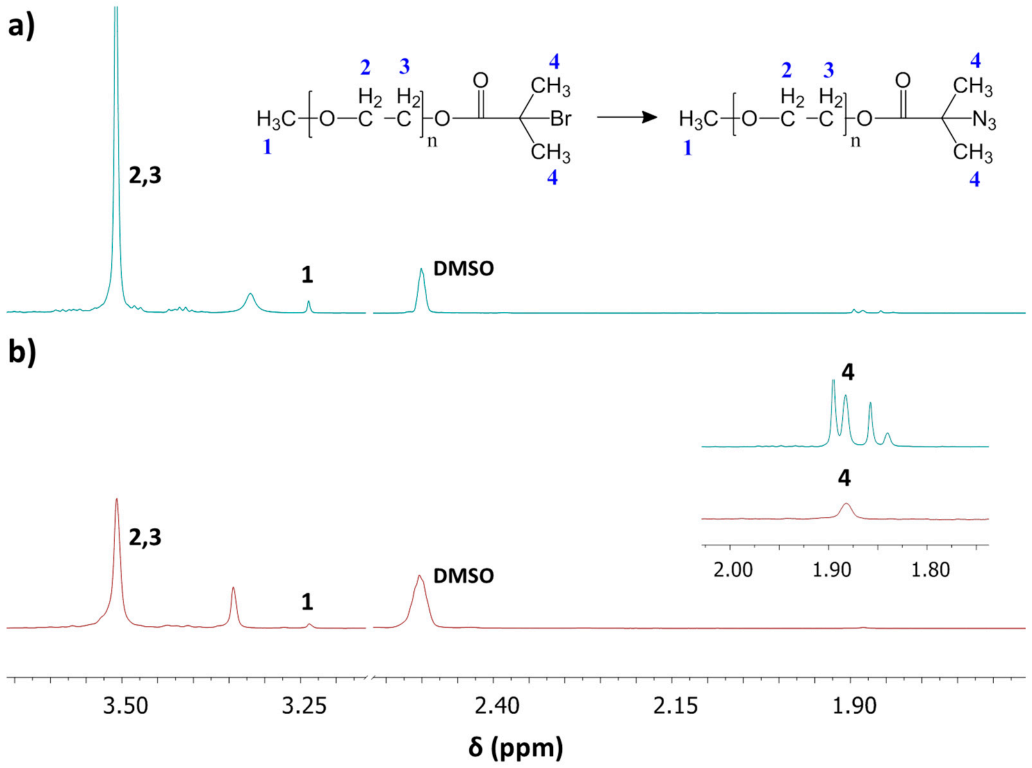

Figure S8. ^{1}H NMR spectra of a) PEG-Br, b) PEG-N_3.

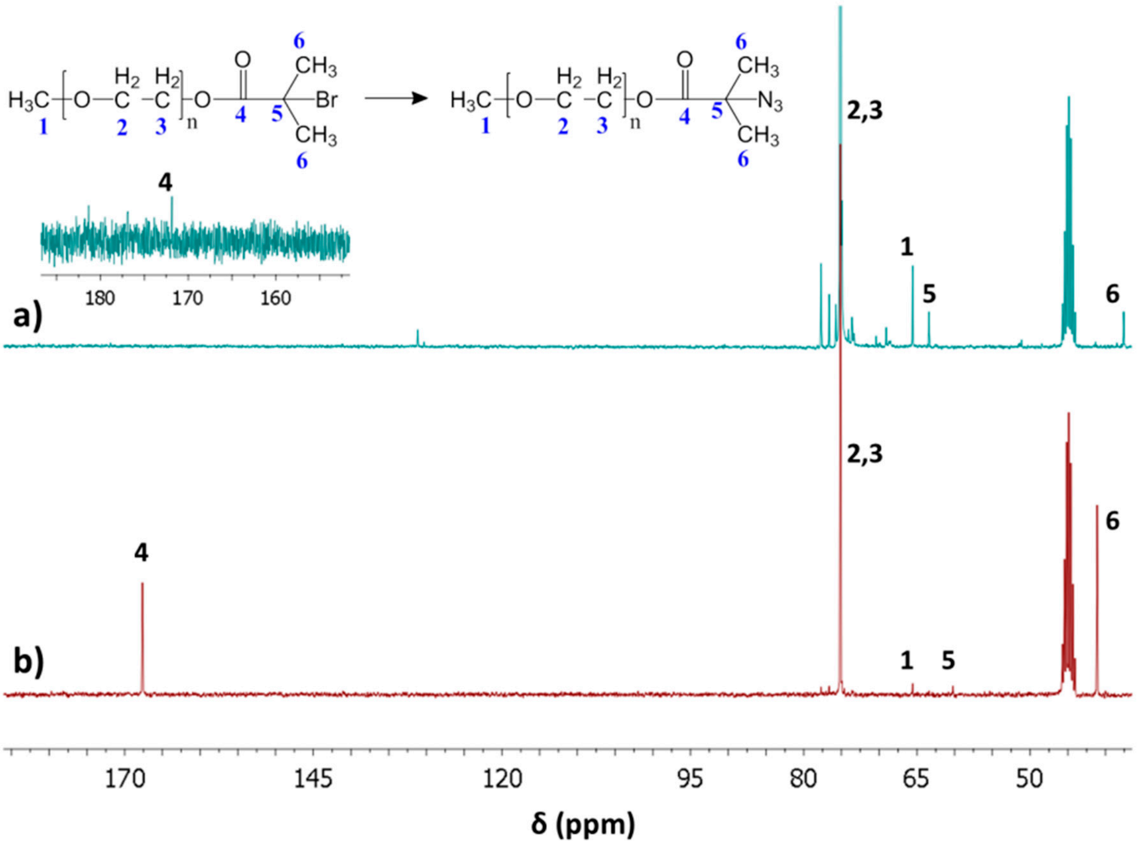

Figure S9. ^{13}C NMR spectra of a) PEG-Br, b) PEG-N_3.

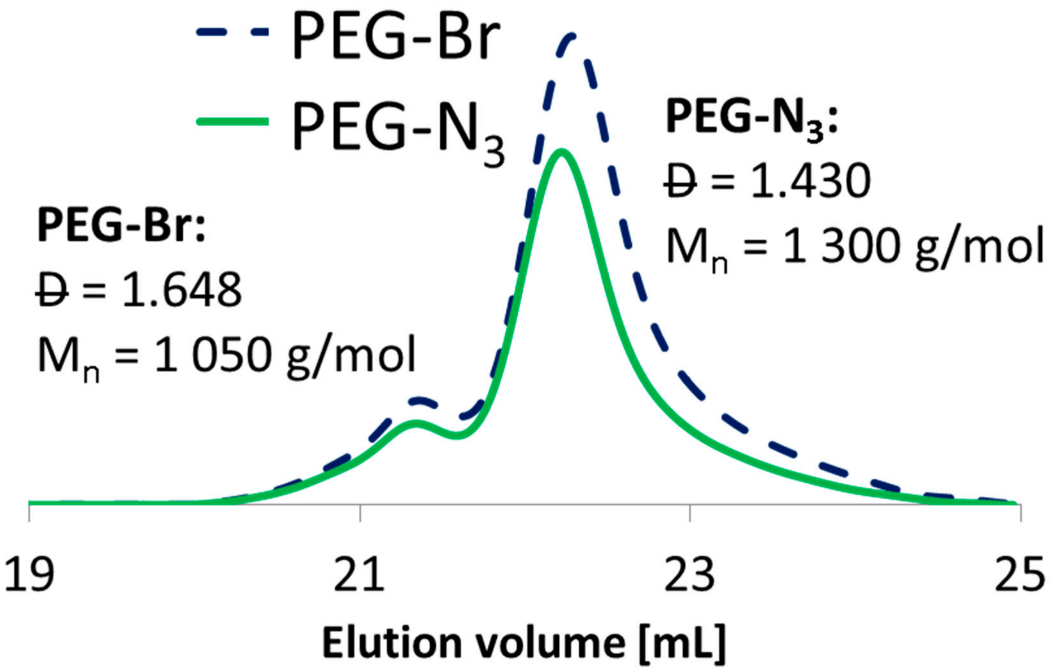

Figure S10. GPC traces of PEG-Br and PEG-N_3.

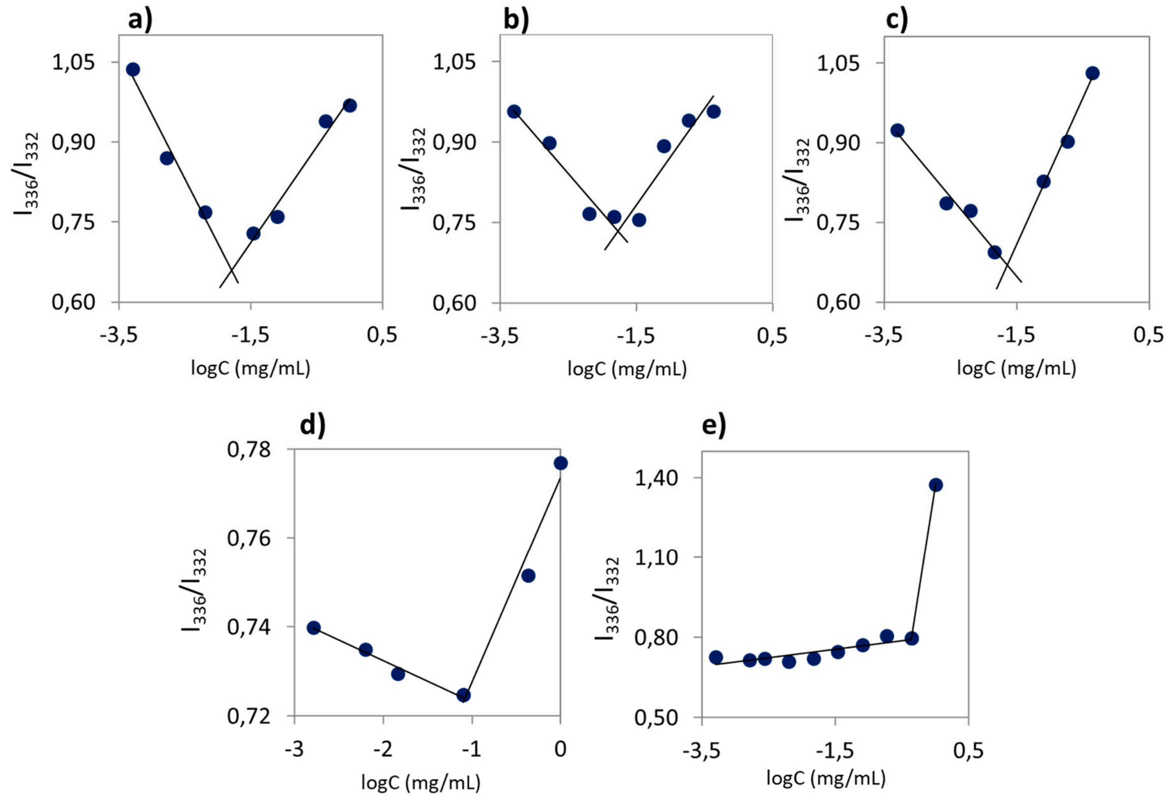

Figure S11. Plots of intensity I_{336}/I_{332} ratio as a function of the logarithm of copolymers concentration in aqueous solution for series **Ic-IIIc** (a-c), and RET series **IVc**, **VIc** (d-e).

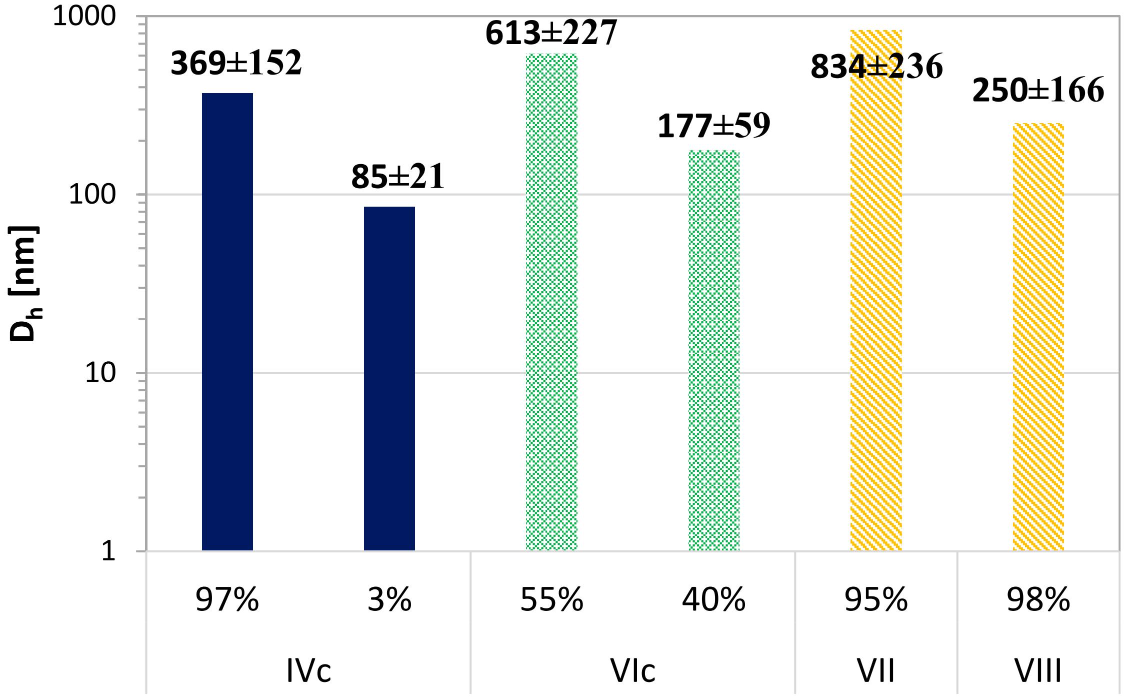

Figure S12. Particle size distribution data for VitC loaded micellar systems based on intensity calculation method.

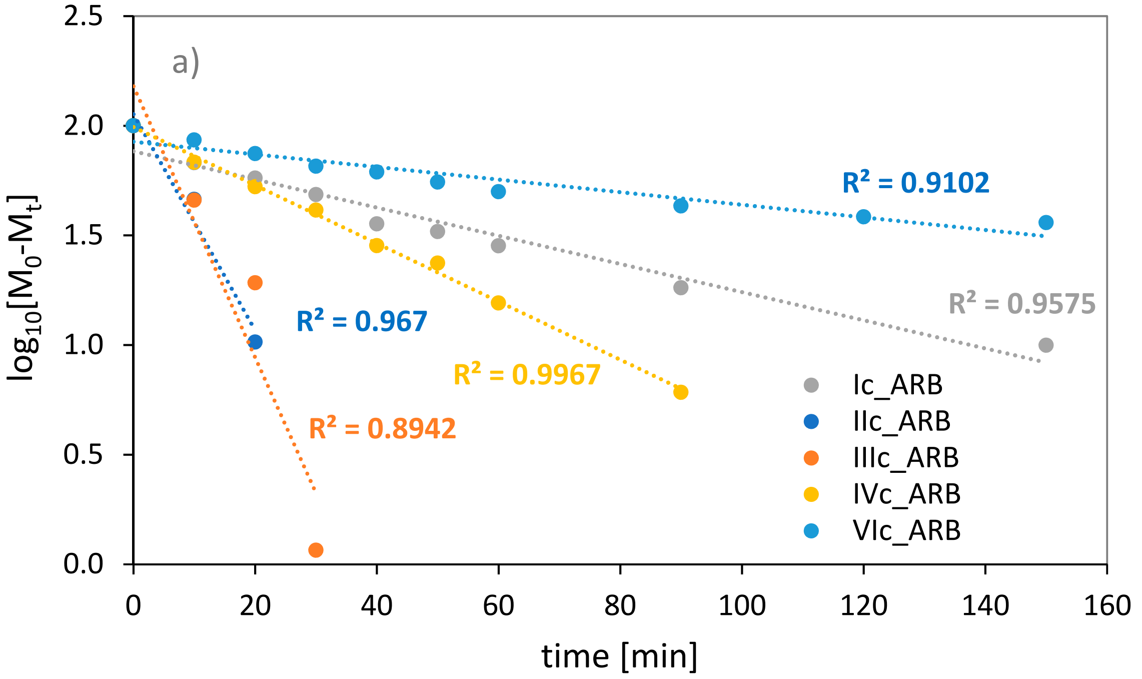

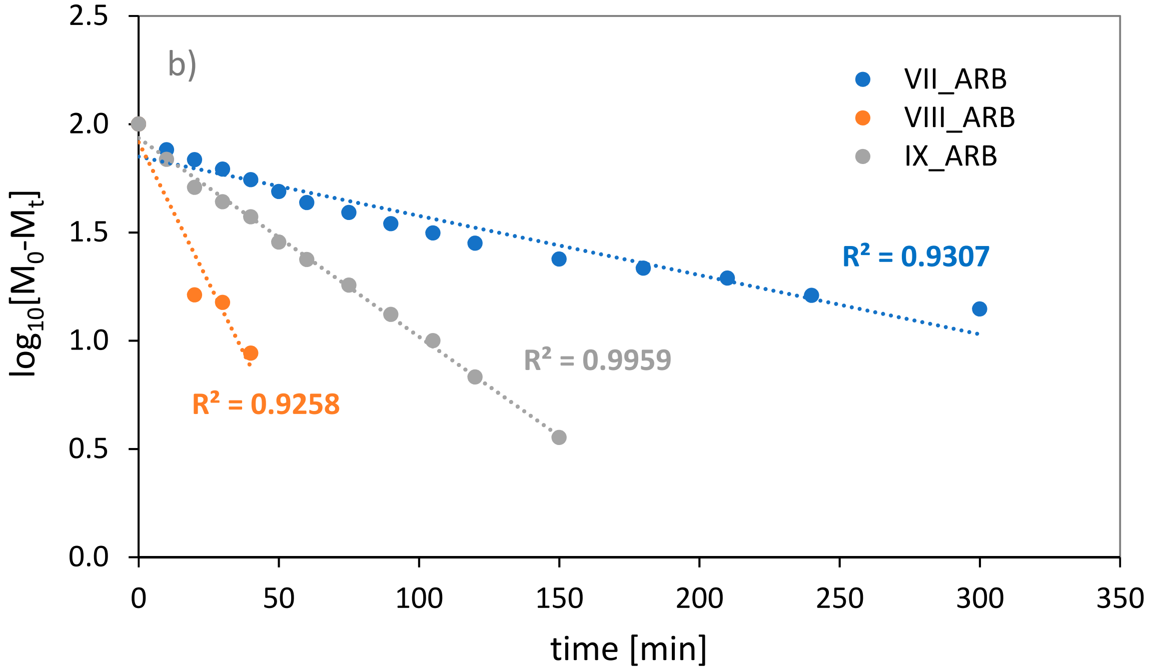

Figure S13. First order kinetic model of ARB release: (a) graft copolymers (b) linear copolymers.

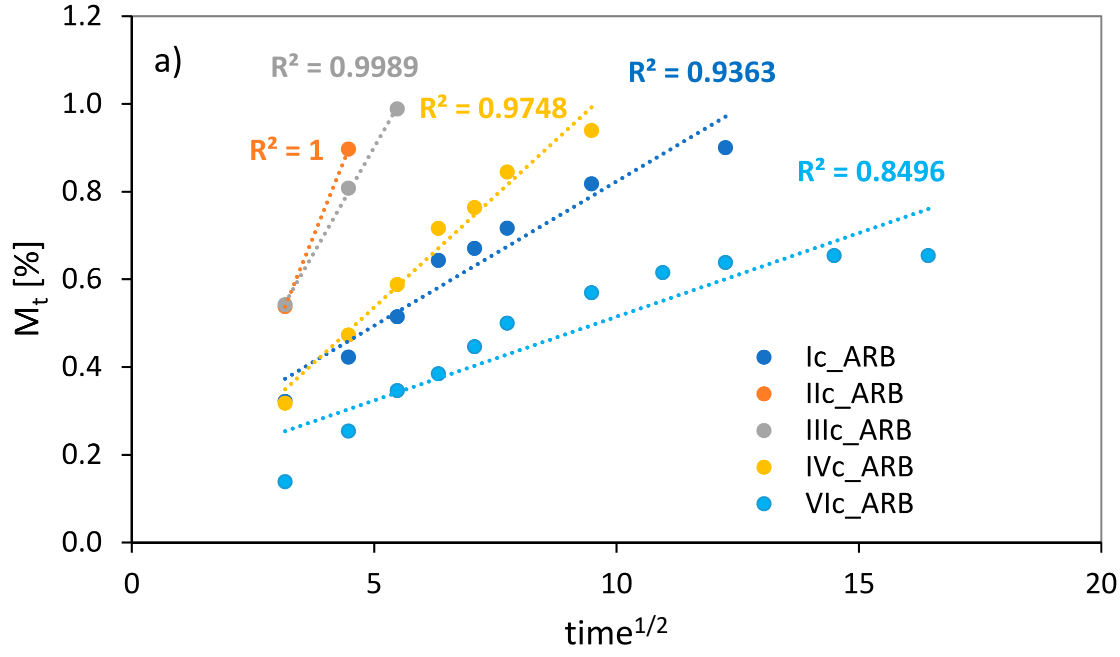

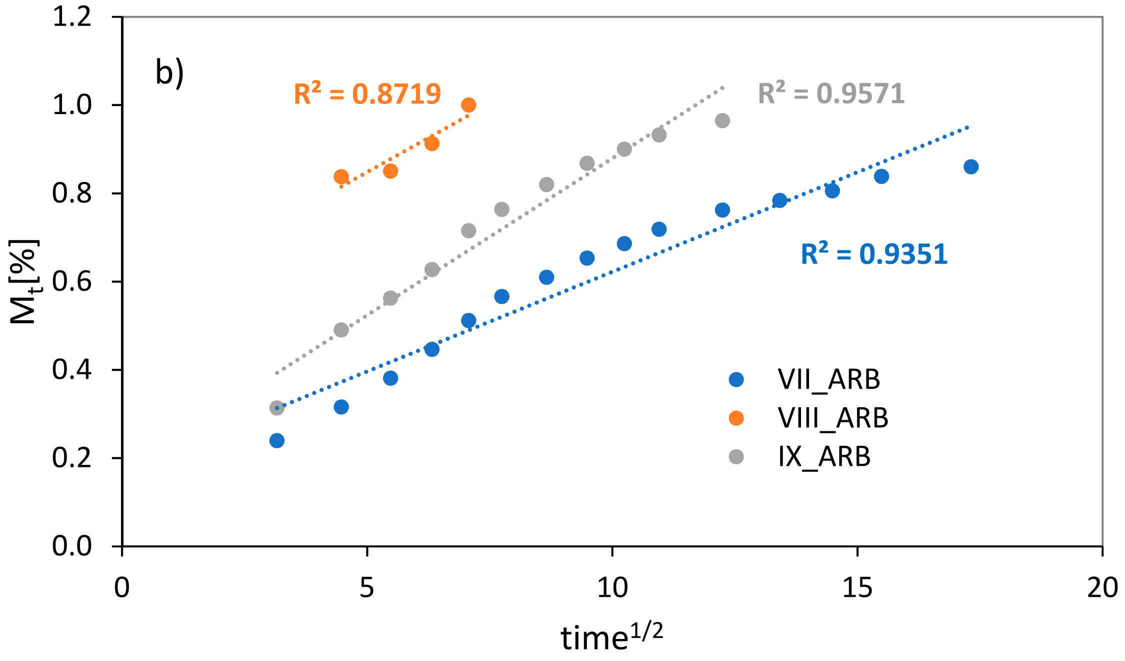

Figure S14. Higuchi kinetic model of ARB release: (a) graft copolymers (b) linear copolymers.

© 2019 by the authors. Submitted for possible open access publication under the terms and conditions of the Creative Commons Attribution (CC BY) license (http://creativecommons.org/licenses/by/4.0/).
